# Cerebral blood flow and cerebrovascular reactivity are modified by maturational stage and exercise training status during youth

**DOI:** 10.1113/EP091279

**Published:** 2023-09-24

**Authors:** Jack S. Talbot, Dean R. Perkins, Christine M. Tallon, Tony G. Dawkins, Andrew J. M. Douglas, Ryan Beckerleg, Andrew Crofts, Melissa E. Wright, Saajan Davies, Jessica J. Steventon, Kevin Murphy, Rachel N. Lord, Christopher J. A. Pugh, Jon L. Oliver, Rhodri S. Lloyd, Philip N. Ainslie, Ali M. McManus, Mike Stembridge

**Affiliations:** ^1^ Cardiff School of Sport and Health Sciences Cardiff Metropolitan University Cardiff UK; ^2^ Centre for Health, Activity and Wellbeing Research Cardiff Metropolitan University Cardiff UK; ^3^ Department of Sport Science University of Innsbruck Innsbruck Austria; ^4^ Centre for Heart, Lung and Vascular Health, School of Health and Exercise Sciences University of British Columbia Okanagan Kelowna Canada; ^5^ Cardiff University Brain Research Imaging Centre (CUBRIC), School of Physics and Astronomy Cardiff University Cardiff UK; ^6^ Youth Physical Development Centre Cardiff Metropolitan University Cardiff UK; ^7^ Sports Performance Research Institute New Zealand AUT University Auckland New Zealand; ^8^ Centre for Sport Science and Human Performance Waikato Institute of Technology Waikato New Zealand

**Keywords:** cerebral perfusion, hypercapnia, maturation, paediatric exercise physiology

## Abstract

Global cerebral blood flow (gCBF) and cerebrovascular reactivity to hypercapnia ($\mathrm{CV}R_{CO_{2}}$) are modulated by gonadal hormone activity, while insulin‐like growth factor 1 facilitates exercise‐mediated cerebral angiogenesis in adults. Whether critical periods of heightened hormonal and neural development during puberty represent an opportunity to further enhance gCBF and $\mathrm{CV}R_{CO_{2}}$ is currently unknown. Therefore, we used duplex ultrasound to assess gCBF and $\mathrm{CV}R_{CO_{2}}$ in *n* = 128 adolescents characterised as endurance‐exercise trained (males: *n* = 30, females: *n* = 36) or untrained (males: *n* = 29, females: *n* = 33). Participants were further categorised as pre‐ (males: *n* = 35, females: *n* = 33) or post‐ (males: *n* = 24, females: *n* = 36) peak height velocity (PHV) to determine pubertal or ‘maturity’ status. Three‐factor ANOVA was used to identify main and interaction effects of maturity status, biological sex and training status on gCBF and $\mathrm{CV}R_{CO_{2}}$. Data are reported as group means (SD). Pre‐PHV youth demonstrated elevated gCBF and slower $\mathrm{CV}R_{CO_{2}}$ mean response times than post‐PHV counterparts (both: *P* ≤ 0.001). gCBF was only elevated in post‐PHV trained males when compared to untrained counterparts (634 (43) vs. 578 (46) ml min^−1^; *P* = 0.007). However, $\mathrm{CV}R_{CO_{2}}$ mean response time was faster in pre‐ (72 (20) vs. 95 (29) s; *P* ≤ 0.001), but not post‐PHV (*P* = 0.721) trained youth when compared to untrained counterparts. Cardiorespiratory fitness was associated with gCBF in post‐PHV youth (*r* ^2^ = 0.19; *P* ≤ 0.001) and $\mathrm{CV}R_{CO_{2}}$ mean response time in pre‐PHV youth (*r* ^2^ = 0.13; *P* = 0.014). Higher cardiorespiratory fitness during adolescence can elevate gCBF while exercise training during childhood primes the development of cerebrovascular function, highlighting the importance of exercise training during the early stages of life in shaping the cerebrovascular phenotype.

## Abstract (highlights)

- **What is the central question of this study?**
  Gonadal hormones modulate cerebrovascular function while insulin‐like growth factor 1 (IGF‐1) facilitates exercise‐mediated cerebral angiogenesis; puberty is a critical period of neurodevelopment alongside elevated gonadal hormone and IGF‐1 activity: but whether exercise training across puberty enhances cerebrovascular function is unkown.
- **What is the main finding and its importance?**
  Cerebral blood flow is elevated in endurance trained adolescent males when compared to untrained counterparts. However, cerebrovascular reactivity to hypercapnia is faster in trained vs. untrained children, but not adolescents. Exercise‐induced improvements in cerebrovascular function are attainable as early as the first decade of life.

## 1 INTRODUCTION

Global cerebral blood flow (gCBF) is tightly regulated by feed‐back and feed‐forward pathways to ensure the close coupling of oxygen delivery to the metabolic demands of the brain (Iadecola, 2017). Puberty coincides with critical periods of neurodevelopment and heightened plasticity for cerebral structures across childhood and adolescence (Ismail et al., 2017), with metabolic demand and gCBF highest during mid‐childhood (Goyal et al., 2014; Paniukov et al., 2020), before declining in a brain region‐specific manner during adolescence (Giedd et al., 1999; Satterthwaite et al., 2014; Tamnes et al., 2017) due to neuronal network alterations such as synaptic pruning (Huttenlocher, 1979) and increased axon myelination (Kwon et al., 2020). While the decline in gCBF across adolescence is a normal physiological characteristic of neural development, a greater magnitude of decline in gCBF during adolescence has been related to increased obesity (Kuzawa & Blair, 2019) and cardiovascular disease risk (Schmithorst et al., 2021).

Endurance exercise training has often (Ainslie et al., 2008; Alfini et al., 2019; Bailey et al., 2013; Chapman et al., 2013; Kleinloog et al., 2019; Tarumi et al., 2013; Thomas et al., 2013), but not always (Kleinloog et al., 2019; Murrell et al., 2013), been shown to increase resting cerebral blood flow or blood velocities in healthy adults. Exercise‐mediated increases in cerebral angiogenesis and neurogenesis are, in‐part, facilitated by insulin‐like growth factor 1 (IGF‐1) activity in adult rats (Lopez‐Lopez et al., 2004; Trejo et al., 2001). Furthermore, cerebral perfusion and cerebrovascular function are modulated by fluctuations in gonadal hormones and their role in signalling endothelial nitric oxide synthase activity (Caulin‐Glaser et al., 1997; Cote et al., 2021; Hutchison et al., 1997; Krause et al., 2006). However, far less is known about the relationship between aerobic fitness, gCBF and cerebrovascular function during childhood and adolescence (Ainslie & McManus, 2016), despite the sex‐specific influx of gonadal hormones and IGF‐1 during puberty (Cole et al., 2015).

Elevated aerobic fitness (${\dot{V}}_{O_{2}\max}$) has been associated with increased cerebral perfusion in pre‐pubertal children (Chaddock‐Heyman et al., 2016). However, the modest relationship was limited to the hippocampus, and was far weaker than the relationship between aerobic fitness and middle cerebral artery blood velocity (MCAv) reported in adults (Bailey et al., 2013). Furthermore, the acute change in MCAv during exercise is attenuated in pre‐pubertal children compared to both adolescents (Weston et al., 2021) and young healthy adults (Ellis et al., 2017; Weston et al., 2021). Indeed, the MCAv response during exercise is related to end‐tidal CO_2_ ($P_{\mathrm{ETC}O_{2}}$) in adults, but not in pre‐pubertal children (Ellis et al., 2017), while the amplitude of cerebrovascular reactivity to CO_2_ ($\mathrm{CV}R_{CO_{2}}$) may increase with chronological age in youths (Leung et al., 2016), and the blood flow response time (τ) to hypercapnia is blunted in children compared to adults (Tallon et al., 2020, 2022). The attenuated $\mathrm{CV}R_{CO_{2}}$ response in children may relate to immature endothelial function due to the relative absence of gonadal hormones (Caulin‐Glaser et al., 1997; Cote et al., 2021; Hutchison et al., 1997; Krause et al., 2006). However, the higher resting gCBF in children may also facilitate a faster partial pressure of arterial CO_2_ ($P_{\mathrm{aC}O_{2}}$) washout (Hoiland et al., 2018). While chronic endurance training appears to influence $\mathrm{CV}R_{CO_{2}}$ in adults (Bailey et al., 2013; Barnes et al., 2013; DuBose et al., 2022; Intzandt et al., 2020; Murrell et al., 2013, 2013), the impact of chronic endurance training on $\mathrm{CV}R_{CO_{2}}$ has yet to be explored in children and adolescents.

The aim of this study was to investigate the impact of exercise training status at different stages of maturation on gCBF and $\mathrm{CV}R_{CO_{2}}$ in males and females. We used a somatic measure of maturity (predicted age at peak height velocity (PHV)) to investigate the role of maturity status, biological sex and training status in males and females (Baxter‐Jones et al., 2005). We hypothesised that: (1) post‐PHV endurance trained youth would demonstrate elevated gCBF compared to their untrained counterparts, whereas there would be no training related differences in pre‐PHV participants; and (2) post‐PHV endurance trained youth would demonstrate a faster $\mathrm{CV}R_{CO_{2}}$ mean response time than untrained counterparts, whereas there would be no training‐related difference in pre‐PHV participants.

## 2 METHODS

### 2.1 Ethical approval

Ethical approval was granted by Cardiff Metropolitan University's School of Sport and Health Sciences Research Ethics Committee (PGR‐1339 and Sta‐3039) and the study conformed to the *Declaration of Helsinki* (2013), except for registration in a database. Detailed, age‐appropriate summaries of the methods and study design were given verbally and in writing to each participant before providing written assent. Furthermore, a legal guardian of each participant was given a verbal and written explanation of the methods and study design before providing written informed consent.

### 2.2 Experimental design

One hundred and seventy‐seven youths volunteered to participate in the study. Participants were excluded if they failed to attend all laboratory visits (*n* = 3) or failed to meet our health and physical activity criteria (*n* = 6). Based on self‐ and parental‐reported physical activity, *n* = 168 participants were categorised as either endurance trained (total: *n* = 90; males: *n* = 42, age = 7.8–18.0 years; females: *n* = 48, age = 8.2–17.0 years) or untrained (total: *n* = 78; males: *n* = 34, age = 8.0–17.7 years; females: *n* = 44, age = 8.0–17.8 years). ‘Trained’ youth had completed ≥3 structured endurance training sessions per week for ≥12 months and were recruited from local endurance‐sport clubs (see Table 1 for training volume data). ‘Untrained’ youth were not taking part in regular exercise or meeting UK Chief Medical Officer's *Physical Activity Guidelines* for children and young people (DHSC, 2019) and were recruited from local schools and community clubs. Following eligibility screening, participants attended the laboratory at Cardiff Metropolitan University on one occasion. Per technical guidelines for the assessment of extra‐cranial gCBF (Thomas et al., 2015), participants refrained from vigorous exercise, caffeine and alcohol for ≥12 h prior to the data collection. Similar to comparable paediatric studies, participants attended the laboratory having fasted for ≥4 h (Hopkins et al., 2015, 2013).

**TABLE 1:** Anthropometric and training status‐related characteristics of participants.

	Pre‐PHV untrained males	Pre‐PHV trained males	Pre‐PHV untrained females	Pre‐PHV trained females	Post‐PHV untrained males	Post‐PHV trained males	Post‐PHV untrained females	Post‐PHV trained females	Maturation status *P‐*value	Sex *P*‐value	Training status *P*‐value	Maturation × Sex interaction *P*‐value	Maturation × Training interaction *P*‐value	Sex × Training interaction *P*‐value	Maturation × Sex × Training interaction *P*‐value
*n*	17	18	15	18	12	12	18	18	–	–	–	–	–	–	–
Maturation offset (years)	−2.7 (1.1)	−2.6 (1.1)	−1.9 (0.9)^b^	−1.7 (0.9)^b^	2.2 (0.9)^a^	2.3 (1.0)^a^	1.9 (1.0)^a^	1.9 (1.0)^a^	**≤0.001**	0.157	0.541	**≤0.001**	0.868	0.998	0.974
Age (years)	10.5 (1.5)	11.1 (1.7)	9.9 (1.3)	10.0 (1.1)^b^	16.4 (1.1)^a^	16.2 (1.2)^a^	14.2 (1.6)^a^, ^b^	14.2 (1.3)^a^, ^b^	**≤0.001**	**≤0.001**	0.574	**0.013**	0.336	0.803	0.602
Body mass (kg)	38.9 (9.8)	34.8 (6.3)	32.6 (6.2)^b^	34.0 (6.3)	63.7 (8.9)^a^	65.6 (10.8)^a^	52.4 (8.0)^a^, ^b^	52.8 (7.9)^a^, ^b^	**≤0.001**	**≤0.001**	0.934	**0.004**	0.378	0.508	0.222
Stature (cm)	145.4 (9.5)	144.2 (8.9)	138.1 (7.8)^b^	140.3 (8.5)	178.5 (7.9)^a^	178.1 (8.4)^a^	161.8 (5.5)^a^, ^b^	164.1 (6.7)^a^, ^b^	**≤0.001**	**≤0.001**	0.613	**≤0.001**	0.859	0.284	0.912
Lean body mass (kg)	29.8 (5.4)	29.7 (5.0)	26.0 (3.9)	27.6 (4.7)	53.4 (5.7)^a^	57.6 (7.7)^a^	40.2 (5.5)^a^, ^b^	42.2 (5.5)^a^, ^b^	**≤0.001**	**≤0.001**	0.052	**≤0.001**	0.236	0.906	0.320
Training volume (h week^−1^)	1.2 (0.8)	7.1 (2.2)^c^	1.2 (0.7)	6.5 (1.8)^c^	0.8 (0.8)	10.1 (2.8)^a^, ^c^	0.5 (0.6)	8.8 (2.7)^a^, ^c^	**0.003**	0.137	**≤0.001**	0.526	**≤0.001**	0.225	0.766
${\dot{V}}_{O_{2}\max}$ (ml min kg LBM^0.93^)	45.7 (7.8)	51.9 (8.8)^c^	40.6 (6.7)^b^	48.1 (5.1)^c^	45.1 (5.4)	55.1 (7.6)^c^	38.1 (6.5)^b^	49.0 (5.4)^b^, ^c^	0.842	**≤0.001**	**≤0.001**	0.382	0.142	0.675	0.930

*Note*: Values are group means (±SD). Bold text indicates *P* ≤ 0.05.

^a^ Significant difference between pre‐ and post‐PHV youths.

^b^ Significant difference between males and females.

^c^ Significant difference between trained and untrained youths.

Abbreviations: LBM, lean body mass; ${\dot{V}}_{O_{2}\max}$, maximal oxygen consumption.

Data collection was conducted in a quiet, temperature‐controlled room with great care to minimise any external sensory stimulation during cerebrovascular measures. Upon arrival, participants completed a series of questionnaires quantifying their weekly endurance training or physical activity levels which were corroborated with parents, before completing anthropometric measurements. Participants were then instructed to lie down in the supine position, where they were fitted with instrumentation for the acquisition of cerebrovascular and cardiorespiratory data. Following baseline measures, we assessed $\mathrm{CV}R_{CO_{2}}$ during a steady‐state hypercapnic challenge previously used in paediatric cohorts (Tallon et al., 2020, 2022). Thirty minutes after the $\mathrm{CV}R_{CO_{2}}$ assessment, cardiorespiratory fitness (${\dot{V}}_{O_{2}\max}$) was then determined via an incremental exercise test to volitional exhaustion and confirmed via a supra‐maximal verification of ${\dot{V}}_{O_{2}\max}$ on the same cycle‐ergometer (Bhammar et al., 2017).

### 2.3 Anthropometrics and estimated maturity status

Body mass (kg) was measured using electronic scales and stature (cm) and sitting height (cm) using a stadiometer, with participants barefoot and wearing light clothing. Anthropometrics, chronological age and sex were used to calculate their ‘maturity offset’ (predicted years from PHV), an estimate of somatic maturation (Mirwald et al., 2002). Participants were classified into pre‐ and post‐PHV groups using ≥0.5 years prior to and post PHV, respectively. To address the study hypotheses, 21 participants were classified as ‘circa‐PHV’ (between −0.5 and 0.5 years from PHV) and excluded from the study due to the standard error associated with the PHV measurement (Mirwald et al., 2002). Skin fold thickness (skin fold callipers, Harpenden, Baty International, Burgess Hill, UK) was assessed at the triceps and sub‐scapular for the estimation of lean body mass (LBM) as previously described (Silva et al., 2013; Slaughter et al., 1988).

### 2.4 Cardiorespiratory monitoring

All cardiorespiratory variables were sampled continuously at 1 kHz via an analog‐to‐digital converter (Powerlab 16/30, ADInstruments Ltd, Oxford, UK) during all resting gCBF and $\mathrm{CV}R_{CO_{2}}$ ultrasound scans. Mean arterial blood pressure (MAP) and heartrate (HR) were measured by finger photoplethysmography (Finometer PRO, Finapres Medical Systems, Amsterdam, The Netherlands). The partial pressure of end‐tidal carbon dioxide ($P_{\mathrm{ETC}O_{2}}$) and oxygen ($P_{\mathrm{ET}O_{2}}$) were sampled via insertion of a sample line into a mouthpiece worn by the participant that connected in series to a bacteriological filter and a calibrated gas analyser (ML206, ADInstruments). All data were interfaced with LabChart (version 8, ADInstruments).

### 2.5 Cerebrovascular measures

Resting measurements of internal carotid (ICA) and vertebral (VA) artery blood flow were acquired following ≥15 min of supine rest. Extra‐cranial artery blood velocity was measured via pulse wave mode concurrently with vessel diameter via B‐mode imaging with a 15 MHz multi‐frequency linear array duplex ultrasound probe (Terason uSmart 3300, Teratech, Burlington, MA, USA) on the right‐hand side of the participant. ICA diameter (ICA_Diam_) and blood velocity were measured at least 1.5 cm distal to the common carotid artery (CCA) bifurcation to eliminate recordings of turbulent and retrograde flow. The VA was measured between C4 and C6 depending on image quality. All vessels were recorded for a minimum of 60 uninterrupted seconds (i.e. without the participant moving, coughing, swallowing or ‘clearing’ their throat). The insonation angle (60°) was unchanged throughout the baseline and $\mathrm{CV}R_{CO_{2}}$ recordings. All recordings were captured and processed in accordance with published guidelines (Thomas et al., 2015). Blood flow (ICA_Q_ and VA_Q_) and shear rate (ICA_SR_ and VA_SR_) in the right‐hand ICA and VA was subsequently calculated as previously described (Black et al., 2008).

(1) Blood flow = peak envelope blood velocity/2 × [*π* × (0.5 × diameter)^2^] × 60
(2) Shear rate = 4 × peak envelope blood velocity/arterial diameter

Resting gCBF was then calculated as: gCBF = 2 × (ICA_Q_ + VA_Q_).

The relative contribution of ICA_Q_ (ICA%_gCBF_) and VA_Q_ (VA%_gCBF_) to gCBF were then calculated. Additionally, cerebrovascular conductance of gCBF (gCBF_CVC_), ICA (ICA_CVC_) and VA (VA_CVC_) were calculated via the following equations:

(1) gCBF_CVC_ = gCBF/MAP
(2) ICA_CVC_ = ICA_Q_/MAP
(3) VA_CVC_ = VA_Q_/MAP

Subsequent Duplex ultrasound scans were completed in 10 participants to calculate the sonographer's coefficient of variation for ICA_Q_ (mean = 4.3%, range = 0.4–9.7%), VA_Q_ (mean = 5.0%, range = 0.9–9.5%) and gCBF (mean = 2.9%, range = 0.2–7.3%). These values fall within the 10% threshold for the group mean coefficient of variation recommended by extracranial duplex ultrasound guidelines (Thomas et al., 2015).

To assess $\mathrm{CV}R_{CO_{2}}$, the participant remained rested in the supine position and wore a mouthpiece attached to a three‐way valve (Hans Rudolph, Shawnee, KS, USA) that was initially open to allow inspiration of ambient room air. Following 1 min of baseline recording, the valve was turned to allow a fixed concentration of 6% inhaled CO_2_, 21% O_2_ and N_2_ balance, administered for 4 min from a 150 litre Douglas bag. At the end of 4 min the valve was turned back to allow the participant to breath ambient room air for 2 min before cessation of the assessment. ICA blood velocity and diameter, $P_{\mathrm{ET}O_{2}}$, $P_{\mathrm{ETC}O_{2}}$, HR and MAP were measured continuously throughout the baseline period and $\mathrm{CV}R_{CO_{2}}$ assessment.

Resting gCBF and $\mathrm{CV}R_{CO_{2}}$ files were visually inspected before analysis. Seven participants did not complete the $\mathrm{CV}R_{CO_{2}}$ protocol. $\mathrm{CV}R_{CO_{2}}$ ultrasound recordings were excluded based on the following criteria: (1) the occurrence of an overt angle change (*n* = 7), (2) excessive movement of the ICA (*n* = 8), (3) overall poor image quality (e.g., blurry ICA walls, *n* = 4) and (4) unacceptable fit of regression model for the determination of $\mathrm{CV}R_{CO_{2}}$ kinetics (*n* = 9). As such, 128 participants were included for resting gCBF analysis and 93 participants were included in the $\mathrm{CV}R_{CO_{2}}$ analysis.

The $\mathrm{CV}R_{CO_{2}}$ baseline values for ICA_Q_, $P_{\mathrm{ET}O_{2}}$, $P_{\mathrm{ETC}O_{2}}$, MAP and HR were calculated during 60 s of supine rest immediately preceding hypercapnia. The $\mathrm{CV}R_{CO_{2}}$ response was interpreted using several approaches, including: averaged ICA_Q_ in the final 30 s of the test (ICA_Q_ during hypercapnia), the difference from baseline ICA_Q_ to ICA_Q_ during hypercapnia (∆ICA_Q_), the percentage change in ICA_Q_ from baseline to hypercapnia (ICA_Q_%) and ICA_Q_% relative to $P_{\mathrm{ETC}O_{2}}$ ($\mathrm{CV}R_{CO_{2}}$) (Skow et al., 2013; Tallon et al., 2022; Willie et al., 2012). Similarly, the difference between baseline values and values during the final 30 s of the test were calculated for HR (∆HR), MAP (∆MAP), $P_{\mathrm{ETC}O_{2}}$ (∆$P_{\mathrm{ETC}O_{2}}$) and $P_{\mathrm{ET}O_{2}}$ (∆$P_{\mathrm{ET}O_{2}}$).

$$\mathrm{CV}R_{CO_{2}}=\left(\left[\Delta \mathrm{IC}A_{Q}/\mathrm{Baseline}\;\mathrm{IC}A_{Q}\right]\times 100\right)/\Delta P_{\mathrm{ETC}O_{2}}$$

### 2.6 Dynamic onset responses to hypercapnia

Pre‐processing included passing 1 Hz ICA_Q_ bins through a median rank of seven filter as previously described (Tallon et al., 2022). Mono‐exponential modelling with a delay term was then used to explore the onset response of ICA_Q_ to hypercapnia using the following equation (GraphPad Prism v.9.0.1; GraphPad Software, Boston, MA, USA):

$$y\left(t\right)=y_{0}+\Delta_{A}(1-e-[\left\{t-\mathrm{TD}\right\}/t])$$

Where *y*(*t*) is the response at a given time; *y*_0_ is the baseline value; Δ_A_ is the baseline corrected absolute change in amplitude from baseline to asymptote; TD is the time delay, allowed to vary in order to optimise the fit; and τ is the time constant of the response (the time taken to reach 63% of the response).

The response to hypercapnia of each participant was modelled from the onset of the 6% CO_2_ stimulus (0 s). Goodness of fit (*r* ^2^ > 0.50) and normality of residuals were used to determine model acceptability. The $\mathrm{CV}R_{CO_{2}}$ mean response time was calculated for ICA_Q_, as:

MRT=TD+τ

### 2.7 Cardiorespiratory fitness

${\dot{V}}_{O_{2}\max}$ was assessed via an incremental exercise test on an electronically braked cycle ergometer (Excalibur Sport, Lode B.V., Gronigen, The Netherlands) to volitional exhaustion. Adjustments were made to the saddle and handlebars of the ergometer for each participant to ensure a comfortable cycling position. Oxygen consumption (${\dot{V}}_{O_{2}}$) and HR (RS400, Polar Electro, Kemple, Finland) were assessed at rest and continuously throughout the exercise protocol (Oxycon Pro, Jaeger, Hoechberg, Germany). The exercise test implemented a ramp incremental protocol where workload increments were determined by participant stature and training status (Perkins et al., 2022). Participants were encouraged to maintain a cadence of 75–85 rpm throughout the protocol. The test was ended once the participant failed to maintain a cadence ≥70 rpm for ≥5 consecutive seconds. Following 15 min of rest, participants completed a constant‐load supramaximal verification test at 105% of power output achieved at peak ${\dot{V}}_{O_{2}}$ during the incremental ramp test to confirm attainment of ${\dot{V}}_{O_{2}\max}$, as recommended for cardiorespiratory fitness testing in paediatric cohorts (Barker et al., 2011; Bhammar et al., 2017). Individual ${\dot{V}}_{O_{2}\max}$ values were then allometrically scaled to LBM using a cohort determined exponent (LBM^0.93^) to account for developmental changes in LBM across youth (Loftin et al., 2016).

### 2.8 Statistical analysis

Power analyses for gCBF data presented in this article were conducted a priori by sampling pilot data assessing MCAv via trans‐cranial Doppler ultrasound in a similar cohort of pre‐ and post‐PHV youth. The minimum required sample size for a statistically significant maturation status, biological sex and training status interaction effect was *n* = 12 per group based on 95% power at a two‐sided 0.05 significance level. As such, we aimed to recruit at least 12 participants in each group to achieve statistical power for main and interaction effects, as well as allowing for data drop‐out due to poor image quality during the $\mathrm{CV}R_{CO_{2}}$ assessment. Statistical analysis was conducted on SPSS Statistics software package (Version 23.0, IBM Corp., Armonk, NY, USA). Normal distributions of outcome variables were confirmed via Shapiro–Wilk statistical tests and visual inspection of *p–p* plots. All data are presented as group means (±SD) with statistical significance set to *P* < 0.05 unless otherwise stated. A three‐factor analysis of variance (ANOVA) was used to determine the main effects of maturity status, biological sex and training status, as well as the interaction effect of these variables on gCBF and $\mathrm{CV}R_{CO_{2}}$. *Post hoc* comparisons were conducted to identify significant differences among groups when significant main or interaction effects were observed. A Bonferroni correction was applied to all post hoc t‐tests to account for multiple comparisons, with adjusted P‐values reported. The main aim of this study was to understand the influence of training status on gCBF and $\mathrm{CV}R_{CO_{2}}$ during different stages of maturity. As such, the reporting of *post hoc* comparisons will focus on the effect of training status on gCBF and $\mathrm{CV}R_{CO_{2}}$. Additionally, linear regression analysis was conducted to quantify the relationship between cardiorespiratory fitness (${\dot{V}}_{O_{2}\max}$), gCBF and $\mathrm{CV}R_{CO_{2}}$ mean response time in pre‐ and post‐PHV youth by grouping trained and untrained individuals as well as males and females.

## 3 RESULTS

### 3.1 Descriptive characteristics

Post‐PHV youth had a higher maturity‐offset, chronological age, stature, body mass, LBM and MAP than their pre‐PHV counterparts (all *P* ≤ 0.001, Table 1). Additionally, post‐PHV youth demonstrated greater training volumes (*P* ≤ 0.001) compared to their pre‐PHV counterparts (*P* ≤ 0.001), but ${\dot{V}}_{O_{2}\max}$ relative to LBM was similar in pre‐ and post‐PHV youth (*P* = 0.842, Table 1). Endurance trained youth had a higher training volume and ${\dot{V}}_{O_{2}\max}$ compared to untrained youth (all *P* ≤ 0.001, Table 1).

### 3.2 The influence of training status on cerebral blood flow

**FIGURE 1:**
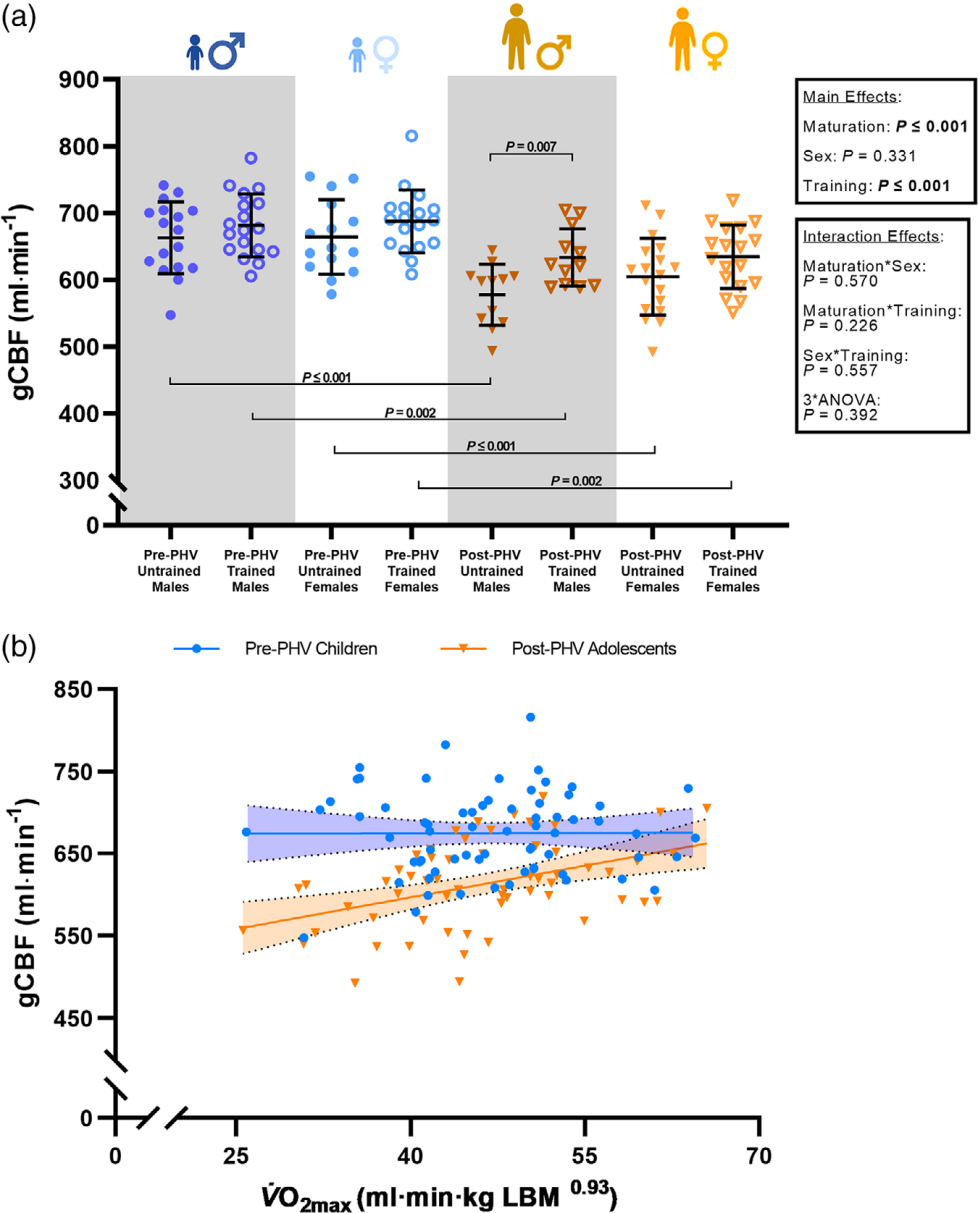
Global cerebral blood flow (gCBF) at rest in males and females (a) and the relationship between peak oxygen uptake allometrically scaled to lean body mass (${\dot{V}}_{O_{2}\max}$) and global cerebral blood flow (gCBF; b) in pre‐ (blue circles) and post‐ (yellow triangles) PHV youth (Pre‐PHV Youth: *R* ^2^ = 0.00; *P* = 0.962. Post‐PHV Youth: *R* ^2^ = 0.19; *P* ≤ 0.001). *P*‐values within the figure plot indicate a significant difference between groups during *post hoc* comparisons.

There was a significant main effect for maturation, with gCBF, ICA_Q_, VA_Q_ and VA%_gCBF_ all lower in post‐PHV youth when compared to pre‐PHV youth (all *P* ≤ 0.001, Table 1 and Figure 1a). However, gCBF, ICA_Q_, VA_Q_, ICA%_gCBF_ and VA%_gCBF_ were similar in males and females (all *P* ≥ 0.05, Table 1 and Figure 1a). Endurance trained youth demonstrated a higher gCBF (*P* ≤ 0.001), ICA_Q_ (*P* = 0.013) and VA_Q_ (*P* = 0.029) when compared to untrained counterparts (Table 1 and Figure 1a). *Post hoc* comparisons revealed that gCBF was lower in post‐PHV untrained males when compared to trained counterparts (−9%; *P* = 0.014), but there were no training differences in pre‐PHV males (−3%; *P* = 0.281), pre‐PHV females (−3%; *P* = 0.181) or post‐PHV females (−5%; *P* = 0.078). Additionally, gCBF was lower in post‐PHV untrained males (−12%; *P* ≤ 0.001) and females (−8%; *P* ≤ 0.001), as well as trained males (−7%; *P* = 0.012) and females (−8%; *P* = 0.002) when compared to their pre‐PHV counterparts. Furthermore, ${\dot{V}}_{O_{2}\max}$ was not associated with gCBF in pre‐PHV youth (*R* ^2^ = 0.00; *P* = 0.962) while ${\dot{V}}_{O_{2}\max}$ was positively associated with gCBF in post‐PHV youth (*R* ^2^ = 0.19; *P* ≤ 0.001). The gradient of the relationship between ${\dot{V}}_{O_{2}\max}$ and gCBF was significantly steeper in post‐PHV youth when compared to pre‐PHV counterparts (*P* = 0.017, Figure 1b).

ICA_Q_ was significantly lower in post‐PHV untrained males when compared to their trained counterparts (−9%; *P* = 0.014), but there were no training differences in pre‐PHV males (0%; *P* = 0.997), pre‐PHV females (−1%; *P* = 0.617) or post‐PHV females (−5%; *P* = 0.071). Additionally, ICA_Q_ was lower in post‐PHV untrained males (−12%; *P* ≤ 0.001) and females (−8%; *P* = 0.012) when compared to their pre‐PHV counterparts, but there were no maturity‐related differences in endurance trained males (−4%; *P* = 0.256) and females (−4%; *P* = 0.166). There were no significant *post hoc* training effects on VA_Q_ (all *P* ≥ 0.05) while ICA and VA diameters, blood velocities and shear rates were similar in trained and untrained youth (all *P* ≥ 0.05, Table 2).

**TABLE 2:** Cerebrovascular characteristics of youth included in the global cerebral blood flow analysis.

	Pre‐PHV untrained males	Pre‐PHV trained males	Pre‐PHV untrained females	Pre‐PHV trained females	Post‐PHV untrained males	Post‐PHV trained males	Post‐PHV untrained females	Post‐PHV trained females	Maturation status *P‐*value	Sex *P*‐value	Training status *P*‐value	Maturation × Sex interaction *P*‐value	Maturation × Training interaction *P*‐value	Sex × Training interaction *P*‐value	Maturation × Sex × Training interaction *P*‐value
*n*	17	18	15	18	12	12	18	18	–	–	–	–	–	–	–
ICA_Diam_ (cm)	0.47 (0.03)	0.47 (0.03)	0.45 (0.02)^b^	0.46 (0.03)	0.50 (0.03)^a^	0.50 (0.03)^a^	0.47 (0.03)^b^	0.47 (0.03)^b^	**≤ 0.001**	**≤ 0.001**	0.233	0.132	0.681	0.267	0.668
ICA blood velocity (cm s^−1^)	47.8 (6.0)	47.9 (6.1)	50.6 (7.4)^b^	49.8 (5.2)	37.1 (3.5)^a^	41.0 (5.1)^a^	45.3 (5.0)^a^, ^b^	46.2 (5.8)^b^	**≤ 0.001**	**≤ 0.001**	0.615	0.102	0.059	0.136	0.988
ICA_SR_ (s^−1^)	406.3 (71.4)	408.1 (75.5)	474.1 (95.4)^b^	431.4 (60.7)	297.0 (38.6)^a^	329.0 (58.7)^a^	390.6 (53.8)^a^, ^b^	395.2 (74.8)^b^	**≤ 0.001**	**≤ 0.001**	0.931	0.167	0.119	0.148	0.728
ICA_Q_ (ml min^−1^)	250.3 (28.1)	250.3 (15.9)	247.8 (23.5)	251.6 (17.6)	219.4 (24.8)^a^	241.2 (15.3)^c^	228.7 (22.6)^a^	241.7 (20.2)	**≤ 0.001**	0.579	**0.013**	0.477	**0.045**	0.743	0.413
ICA_CVC_ (ml min mmHg^−1^)	3.29 (0.47)	3.35 (0.35)	3.43 (0.34)	3.27 (0.40)	2.72 (0.42)^a^	3.00 (0.31)^a^	2.92 (0.33)^a^	3.08 (0.43)	**≤ 0.001**	0.226	0.246	0.422	**0.047**	0.232	0.705
VA diameter (mm)	0.37 (0.02)	0.37 (0.04)	0.36 (0.03)	0.37 (0.03)	0.38 (0.2)	0.39 (0.02)	0.37 (0.03)	0.37 (0.03)	0.123	**0.020**	0.492	0.152	0.644	0.373	0.607
VA blood velocity (cm s^−1^)	24.9 (5.2)	29.3 (7.4)^c^	28.2 (5.3)^b^	29.1 (4.4)	20.2 (1.8)^a^	21.6 (3.7)^a^	24.5 (3.2)^a^, ^b^	23.9 (3.3)^a^	**≤ 0.001**	**0.004**	0.074	0.279	0.173	0.102	0.610
VA_SR_ (s^−1^)	267.7 (53.6)	330.1 (105.4)^c^	319.7 (83.3)^b^	318.5 (62.7)	211.9 (24.7)^a^	223.0 (44.1)^a^	274.0 (45.3)^b^	262.8 (55.3)^a^	**≤ 0.001**	**0.003**	0.202	0.198	0.200	0.074	0.387
VA_Q_ (ml min^−1^)	81.2 (20.8)	90.5 (22.4)	84.4 (12.4)	92.2 (15.8)	69.6 (8.7)	75.7 (13.8)^a^	73.7 (12.9)	75.7 (12.4)^a^	**≤ 0.001**	0.428	**0.029**	0.952	0.425	0.626	0.802
VA_CVC_ (ml min mmHg^−1^)	1.06 (0.27)	1.21 (0.32)	1.17 (0.19)	1.19 (0.23)	0.86 (0.11)^a^	0.94 (0.21)^a^	0.95 (0.20)^a^	0.96 (0.16)^a^	**≤ 0.001**	0.234	0.094	0.924	0.650	0.234	0.756
gCBF (ml min^−1^)	663.1 (53.7)	681.5 (47.1)	664.3 (55.7)	687.6 (47.1)	577.9 (45.7)^a^	633.7 (42.9)^a^, ^c^	604.9 (57.6)^a^	634.6 (47.4)^a^	**≤ 0.001**	0.331	**≤ 0.001**	0.570	0.226	0.557	0.392
gCBF_CVC_ (ml min mmHg^−1^)	8.72 (0.95)	9.11 (0.97)	8.92 (0.85)	8.92 (1.03)	7.16 (0.84)	7.88 (0.91)	7.72 (0.94)	8.08 (1.02)	**≤ 0.001**	0.125	0.086	0.487	0.162	0.128	0.651
ICA%_gCBF_	75.5 (5.6)	73.7 (5.3)	74.6 (3.1)	73.2 (3.7)	75.8 (3.6)	76.2 (3.4)	75.6 (3.1)	76.2 (3.4)	**0.021**	0.617	0.446	0.693	0.157	0.841	0.897
VA%_gCBF_	24.5 (5.6)	26.3 (5.3)	25.4 (3.1)	26.8 (3.7)	24.2 (3.6)	23.8 (3.4)	24.4 (3.1)	23.8 (3.4)	**0.021**	0.617	0.446	0.693	0.157	0.841	0.897
MAP (mmHg)	76 (5)	75 (5)	72 (5)	78 (6)^c^	81 (7)^a^	81 (5)^a^	79 (5)^a^	79 (6)	**≤0.001**	0.148	0.311	0.503	0.323	0.069	0.165
$P_{\mathrm{ETC}O_{2}}$ (mmHg)	38.5 (2.7)	39.6 (3.1)	38.8 (3.0)	38.4 (2.7)	40.4 (2.8)^a^	40.5 (1.5)^a^	39.7 (2.1)^a^	39.3 (2.5)^a^	**0.016**	0.088	0.792	0.363	0.309	0.561	0.316

*Note*: Values are group means (± SD). Bold text indicates *P* ≤ 0.05.

^a^ Significant difference between pre‐ and post‐PHV youths.

^b^ Significant difference between males and females.

^c^ Significant difference between trained and untrained youths.

Abbreviations: gCBF, global cerebral blood flow; gCBF_CVC_, gCBF conductance; ICA, internal carotid artery; ICA_CVC_, ICA conductance; ICA_Diam_, ICA diameter; ICA_Q_, ICA blood flow; ICA_SR_, ICA shear rate; MAP, mean arterial blood pressure; $P_{\mathrm{ETC}O_{2}}$, partial pressure of end‐tidal carbon dioxide; VA, vertebral artery; VA_CVC_, VA conductance; VA_Diam_, VA diameter; VA_SR_, VA shear rate; VA_Q_, VA blood flow; ICA%_gCBF_, ICA contribution to gCBF; VA%_gCBF_, VA contribution to gCBF.

### 3.3 The influence of training status on steady‐state cerebrovascular reactivity to carbon dioxide

**FIGURE 2:**
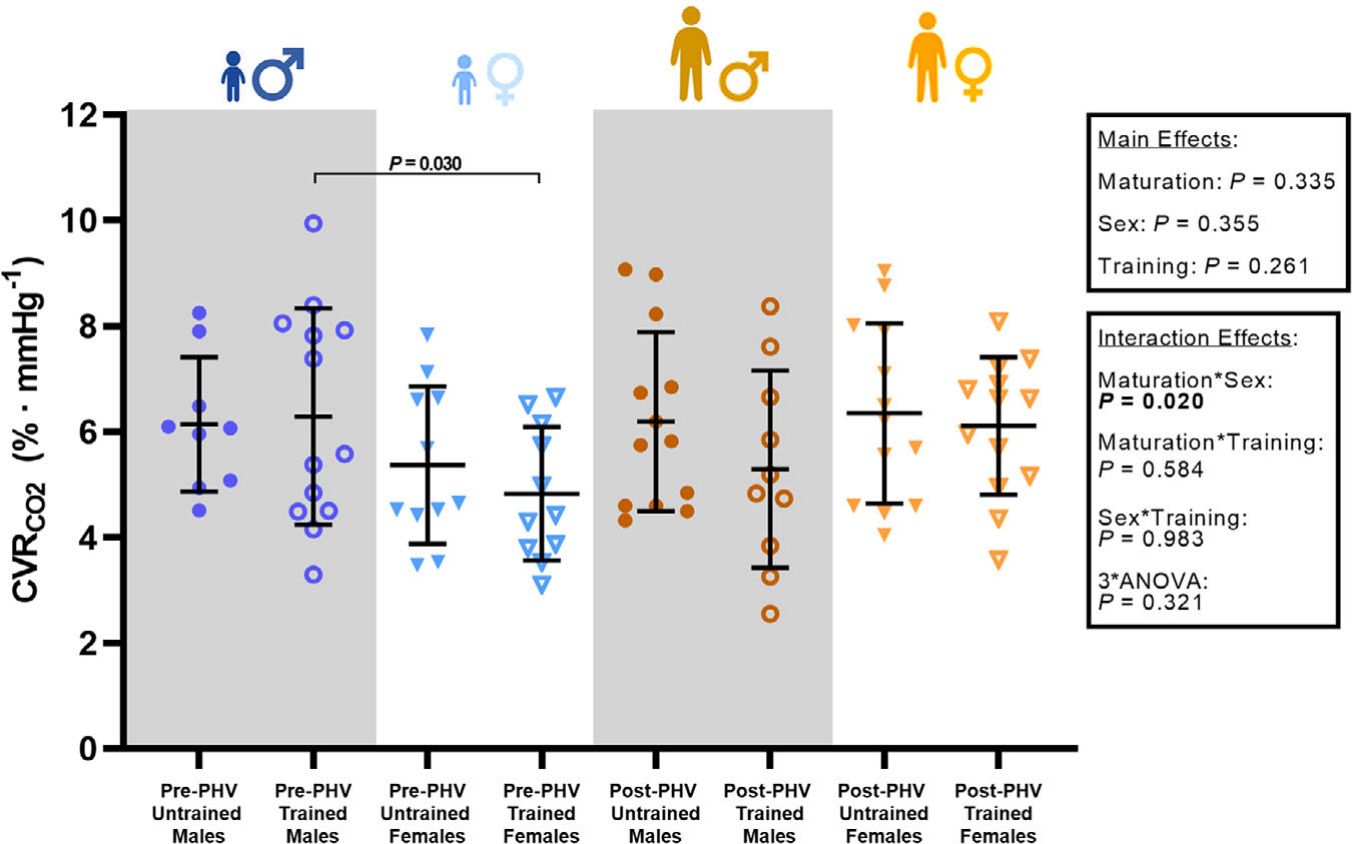
The percentage change in internal carotid artery blood flow relative to the change in $P_{\mathrm{ETC}O_{2}}$ in males and females (steady‐state $\mathrm{CV}R_{CO_{2}}$). *P*‐values within the figure plot indicate a significant difference between groups during *post hoc* comparisons.

During the $\mathrm{CV}R_{CO_{2}}$ stimulus, pre‐PHV youth demonstrated a similar *∆* $P_{\mathrm{ETC}O_{2}}$ (*P* = 0.661), *∆* $P_{\mathrm{ET}O_{2}}$ (*P* = 0.152), *∆*MAP (*P* = 0.604), ΔICA_Diam_ (*P* = 0.627) and ΔICA_CVC_ (*P* = 0.768) to their post‐PHV counterparts (Table 3). Likewise, males demonstrated a similar *∆* $P_{\mathrm{ETC}O_{2}}$ (*P* = 0.076), *∆* $P_{\mathrm{ET}O_{2}}$ (*P* = 0.683), *∆*MAP (*P* = 0.077), ΔICA_Diam_ (*P* = 0.332) and ΔICA_CVC_ (*P* = 0.684) to females. However, the ∆HR was higher in pre‐PHV youth when compared to post‐PHV youth (*P* ≤ 0.001) and lower in males when compared to females (*P* = 0.003). The ΔICA_Q_ was lower in post‐PHV trained males when compared to pre‐PHV trained males (*P* = 0.032) and post‐PHV trained females (*P* = 0.032). There were no maturity‐related differences in $\mathrm{CV}R_{CO_{2}}$ (*P* = 0.335, Figure 2), but $\mathrm{CV}R_{CO_{2}}$ was higher in pre‐PHV trained males when compared to female counterparts (*P* = 0.030, Figure 2).

**TABLE 3:** The change in cerebrovascular and respiratory measures in youth during cerebrovascular reactivity to carbon dioxide.

	Pre‐PHV untrained males	Pre‐PHV trained males	Pre‐PHV untrained females	Pre‐PHV trained females	Post‐PHV untrained males	Post‐PHV trained males	Post‐PHV untrained females	Post‐PHV trained females	Maturation status *P‐*value	Sex *P*‐value	Training status *P*‐value	Maturation × Sex interaction *P*‐value	Maturation × Training interaction *P*‐value	Sex × Training interaction *P*‐value	Maturation × Sex × Training interaction *P*‐value
*n*	9	13	11	11	13	10	13	13	–	–	–	–	–	–	–
∆ICA_Q_ (ml min^−1^)	134.5 (45.4)	133.7 (43.3)	131.9 (56.8)	106.7 (29.2)	115.6 (30.3)	94.7 (26.2)^a^	129.7 (49.4)	131.8 (31.7)^b^	0.304	0.525	0.189	**0.019**	0.832	0.968	0.165
∆ICA_Diam_ (cm)	0.01 (0.01)	0.02 (0.02)	0.02 (0.02)	0.02 (0.02)	0.01 (0.01)	0.01 (0.01)	0.011 (0.01)	0.02 (0.01)	0.627	0.332	0.400	0.862	0.956	0.990	0.265
∆ICA blood velocity (cm s^−1^)	24.3 (10.6)	21.0 (8.5)	22.5 (11.4)	16.4 (4.5)	17.2 (5.2)^a^	13.7 (3.6)^a^	20.4 (6.3)	20.5 (7.8)^b^	0.056	0.567	**0.048**	**0.011**	0.359	0.893	0.315
∆ICA_SR_ (s^−1^)	198.2 (121.6)	161.8 (83.1)	170.1 (116.9)	120.1 (39.2)	126.6 (46.3)^a^	100.0 (26.3)	151.6 (50.9)	161.7 (90.8)	0.094	0.796	0.118	**0.019**	0.286	0.722	0.441
∆ICA_CVC_ (ml min mmHg^−1^)	1.32 (0.47)	1.51 (0.46)	1.36 (0.60)	1.22 (0.46)	1.25 (0.34)	1.17 (0.52)	1.35 (0.57)	1.51 (0.56)	0.768	0.684	0.807	0.146	0.996	0.848	0.222
∆$P_{\mathrm{ETC}O_{2}}$ (mmHg)	9.0 (1.6)	9.2 (1.4)	8.9 (2.1)	9.6 (1.7)	9.3 (1.7)	8.6 (1.8)	9.4 (2.2)	9.7 (0.8)	0.661	0.076	0.410	0.922	0.914	0.961	0.144
∆$P_{\mathrm{ET}O_{2}}$ (mmHg)	32.7 (4.6)	31.7 (3.2)	30.2 (5.1)	30.8 (3.2)	28.4 (8.2)	26.9 (6.1)	30.0 (5.8)	31.3 (3.3)	0.154	0.683	0.929	0.134	0.993	0.475	0.838
∆HR (b min^−1^)	12 (5)	11 (6)	15 (3)	15 (6)	6 (4)^a^	6 (5)^a^	9 (6)^a^	9 (4)^a^	**≤0.001**	**0.003**	0.927	0.858	0.794	0.990	0.996
∆MAP (mmHg)	4 (4)	5 (5)	4 (4)	4 (3)	4 (4)	2 (3)	5 (4)	6 (3)	0.604	0.077	0.487	0.647	0.346	0.458	0.504

*Note*: Values are group means (±SD). Bold text indicates *P* ≤ 0.05.

^a^ Significant difference between pre‐ and post‐PHV youths.

^b^ Significant difference between males and females.

^c^ Significant difference between trained and untrained youth.

Abbreviations: HR, heart rate; ICA_CVC_, internal carotid artery cerebrovascular conductance; ICA_Diam_, ICA diameter; ICA_Q_, internal carotid artery blood flow; ICA_SR_, internal carotid artery shear rate; MAP, mean arterial pressure; $P_{\mathrm{ETC}O_{2}}$, partial pressure of end‐tidal carbon dioxide; $P_{\mathrm{ET}O_{2}}$, partial pressure of end‐tidal oxygen.

There was no effect of training status on the *∆* $P_{\mathrm{ETC}O_{2}}$ (*P* = 0.410), *∆* $P_{\mathrm{ET}O_{2}}$ (*P* = 0.929), *∆*MAP (*P* = 0.487) or *∆*HR (*P* = 0.927) during the $\mathrm{CV}R_{CO_{2}}$ stimulus (Table 3). Likewise, there were no training status‐related differences in the ΔICA_Diam_ (*P* = 0.400), ΔICA_SR_ (*P* = 0.118) or ΔICA_CVC_ (*P* = 0.807). The ΔICA blood velocity was lower in endurance trained youth compared to untrained youth (*P* = 0.048). However, there were no differences in the ΔICA blood velocity during *post hoc* comparisons of trained and untrained groups (all *P* ≥ 0.05). Additionally, there was no effect of training status on the ΔICA_Q_ (*P* = 0.189) or $\mathrm{CV}R_{CO_{2}}$ (*P* = 0.261, Figure 2).

### 3.4 The influence of training status on cerebrovascular mean response time to carbon dioxide

**FIGURE 3:**
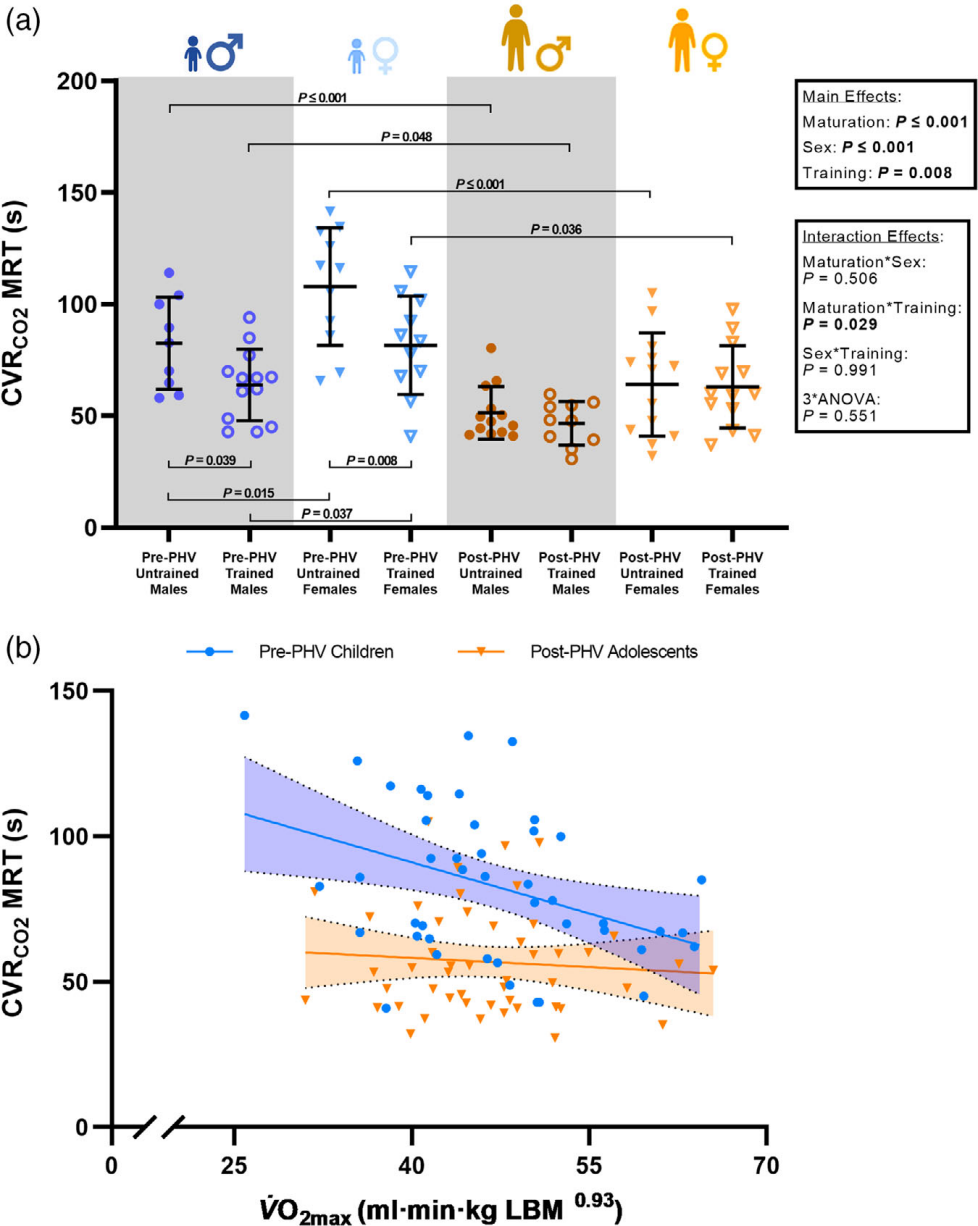
The internal carotid artery blood flow mean response time ($\mathrm{CV}R_{CO_{2}}$ MRT) in males and females during cerebrovascular reactivity to carbon dioxide (a) and the relationship between peak oxygen uptake allometrically scaled to lean body mass (${\dot{V}}_{O_{2}\max}$) and $\mathrm{CV}R_{CO_{2}}$ MRT (b) in pre‐ (blue circles) and post‐ (yellow triangles) PHV youth (Pre‐PHV Youth: *R* ^2^ = 0.13; *P* = 0.014. Post‐PHV Youth: *R* ^2^ = 0.02; *P* = 0.406). *P*‐values within the figure plot indicate a significant difference between groups during *post hoc* comparisons.

During the $\mathrm{CV}R_{CO_{2}}$ stimulus the $P_{\mathrm{ETC}O_{2}}$ mean response time was similar in pre‐ and post‐PHV youth (24 ± 7 vs. 27 ± 8 s; *P* = 0.059). However, $P_{\mathrm{ETC}O_{2}}$ mean response time was faster in males when compared to females (23 ± 7 vs. 28 ± 8 s; *P* = 0.005). The ICA_Q_ mean response time ($\mathrm{CV}R_{CO_{2}}$ mean response time) was slower in pre‐PHV youth compared to post‐PHV youth (*P* ≤ 0.001) and faster in males when compared to females (*P* ≤ 0.001, Figure 3a).

The $P_{\mathrm{ETC}O_{2}}$ mean response time was similar in endurance trained and untrained youth (24 ± 7 vs. 27 ± 8 s; *P* = 0.076). The ICA_Q_ mean response time was faster in endurance trained youth when compared to untrained counterparts (*P* = 0.008, Figure 3). *Post hoc* comparisons revealed that the ICA_Q_ mean response time was 23% faster in pre‐PHV trained males (*P* = 0.039) and 22% faster in pre‐PHV trained females (*P* = 0.008) when compared to their untrained counterparts (Figure 3). However, there were no training‐related differences in post‐PHV males (*P* = 0.585) or females (*P* = 0.951). Furthermore, the ICA_Q_ mean response time was 27% slower in pre‐PHV trained males (*P* = 0.048), 38% slower in pre‐PHV untrained males (*P* ≤ 0.001), 22% slower in pre‐PHV trained females (*P* = 0.036) and 40% slower in pre‐PHV untrained females (*P* ≤ 0.001) when compared to their post‐PHV counterparts (Figure 3a). Furthermore, ${\dot{V}}_{O_{2}\max}$ was positively associated with $\mathrm{CV}R_{CO_{2}}$ mean response time in pre‐PHV youth (*R* ^2^ = 0.13; *P* = 0.014) but not post‐PHV youth (*R* ^2^ = 0.02; *P* = 0.406, Figure 3b).

## 4 DISCUSSION

The aim of this study was to investigate the impact of training status at different stages of maturation on gCBF and $\mathrm{CV}R_{CO_{2}}$ in males and females. For the first time, we report that: (1) endurance trained adolescents demonstrate higher gCBF, but only in post‐PHV males with no training effects in younger groups or females; and (2) pre‐PHV endurance trained males and females demonstrate faster $\mathrm{CV}R_{CO_{2}}$ mean response times than their untrained counterparts, whereas there were no training related differences in post‐PHV youth. Collectively, our data indicate that endurance training can elevate resting cerebral blood flow following the onset of pubertal development in males, while cerebrovascular reactivity to CO_2_ appears to be malleable in males and females at an earlier age. Our findings highlight the importance of exercise across the maturational spectrum.

### 4.1 Endurance training status elevates cerebral blood flow in post‐PHV males only

We have demonstrated lower gCBF in post‐PHV males and females when compared to pre‐PHV counterparts, highlighting that the trajectory of gCBF during youth may be impacted by key developmental changes during somatic maturation, rather than chronological age (Satterthwaite et al., 2014; Wu et al., 2016). Furthermore, cerebral blood flow was similar in males and females across maturity stages, despite previous reports of divergent trajectories in males and females across youth (Lenroot & Giedd, 2010; Satterthwaite et al., 2014; Vandekar et al., 2019). Accordingly, sex differences in cerebral blood flow across youth may dissipate when controlling for sex‐specific trajectories in somatic maturation. The relative contributions of anterior (ICA_Q_) and posterior (VA_Q_) cerebral blood flow to global cerebral blood flow also provided novel insight into the developmental trajectory of cerebral perfusion. Posterior cerebral blood flow (−15%) experienced a larger decline than anterior cerebral blood flow (−7%) from pre‐ to post‐PHV, resulting in a larger relative contribution of anterior blood flow to global cerebral blood flow in post‐PHV youth when compared to pre‐PHV counterparts. Taken together, these observations suggest that the decline in cerebral metabolism during youth is not localised to one specific region, but there is regional heterogeneity in the magnitude of the decline in cerebral perfusion which may be explained by further development of anterior brain regions during adolescence (Casey et al., 2000). Although the range in the coefficient of variation for gCBF, ICA_Q_ and VA_Q_ reported in this manuscript suggests that there is reasonable inter‐individual variability in the reliability of duplex ultrasound to measure cerebral blood flow, the maturity‐related group differences reported in the manuscript exceed the sonographer's average coefficient of variation (see Methods). Therefore, our data support the notion that gCBF declines across youth, potentially due to changes in cerebral metabolism brought about by a reduction in neuronal synapses and myelination of neuronal axons (Kwon et al., 2020).

Training‐related adaptations in cerebral blood flow were most apparent in the anterior cerebral circulation of post‐PHV males. gCBF and ICA_Q_ were higher in post‐PHV trained males compared to untrained counterparts – with group differences beyond the sonographer's average coefficient of variation – while there were no training‐related differences in females across youth. As such, the anterior cerebral circulation may benefit from endurance training more than the posterior circulation during adolescence, particularly in older male adolescents. This anterior–posterior heterogeneity may reflect further potential for exercise‐mediated adaptations in brain regions that develop later during adolescence, such as the pre‐frontal cortex (Casey et al., 2000), which also demonstrate greater activation during acute exercise (Yanagisawa et al., 2010). Furthermore, the significant relationship between cardiorespiratory fitness and gCBF in post‐PHV youth in the current study, despite lower gCBF with somatic maturity, reinforces that the positive influence that cardiorespiratory fitness has on resting gCBF is only attainable following critical periods of neurodevelopment. That said, given the range in the coefficient of variation for duplex assessments of cerebral blood flow across individuals (see Methods), the efficacy of exercise training to elevate cerebral blood flow in adolescents may be somewhat overstated by the imaging techniques used in the current study.

The lack of training‐related differences in cerebral blood flow in pre‐PHV may be explained by the blunted cerebral blood flow response to acute exercise observed when compared to adults (Ellis et al., 2017). The relatively smaller change in cerebral blood flow in pre‐PHV children during acute exercise likely translates to a diminished cerebrovascular shear stress stimulus. Consequently, there may be an attenuated expression of hormones and proteins like IGF‐1, vascular endothelial growth factor and brain‐derived neurotrophic factor, resulting in limited promotion of cerebrovascular angiogenesis and neurogenesis (Lopez‐Lopez et al., 2004; Punglia et al., 1997; Trejo et al., 2001). Likewise, given that IGF‐1 has increased expression during somatic maturation (Löfqvist et al., 2001), chronic exercise‐mediated adaptations in cerebral blood flow may be further limited prior to somatic maturation. There is little evidence to suggest that this maturity‐related milestone in the efficacy of endurance training to alter resting gCBF has negative implications for neurovascular function during adolescence. However, understanding the influence of exercise training on cerebrovascular function in response to neural and metabolic stimuli during this critical period of neural development, rather than just resting haemodynamics, may have more sensitive implications for long‐term neurovascular function during adulthood (Nyberg et al., 2014).

### 4.2 Maturation, biological sex and training status all modulate cerebrovascular function

Our data suggest that steady‐state cerebrovascular reactivity to CO_2_ is similar in pre‐ and post‐PHV youth. There is some evidence that $\mathrm{CV}R_{CO_{2}}$ increases across youth, before plateauing across early adulthood (Leung et al., 2016). However, Leung et al. (2016) utilised 45 s stages of hypercapnia, which is unlikely to have been long enough to induce steady‐state $\mathrm{CV}R_{CO_{2}}$ (Carr et al., 2021). Herein, we report a slower $\mathrm{CV}R_{CO_{2}}$ mean response time in pre‐PHV youth (82 s) compared to post‐PHV counterparts (57 s). However, the large range in $\mathrm{CV}R_{CO_{2}}$ mean response times for pre‐PHV youth in the current study (43–142 s) suggests that hypercapnia (without targeted clamping of $P_{\mathrm{ET}O_{2}}$) in pre‐PHV youth should last for at least 150 s to accurately characterise developmental changes in $\mathrm{CV}R_{CO_{2}}$ across youth. Biological sex may also modulate steady‐state $\mathrm{CV}R_{CO_{2}}$, with lower values in pre‐PHV females vs. males. We speculate that hormone‐related reductions in cerebrovascular tone associated with an influx of oestrogen in females (Cote et al., 2021) and, conversely, opposing effects of androgenic hormones in males create a divergent developmental trajectory in steady state $\mathrm{CV}R_{CO_{2}}$ across adolescence. Likewise, the gonadal hormone‐mediated capacity for changes in steady‐state $\mathrm{CV}R_{CO_{2}}$ may modulate the kinetics of the response. Males presented a faster $\mathrm{CV}R_{CO_{2}}$ mean response time than females across youth, while resting ICA blood velocities – which were higher in females when compared to males – significantly influenced the $\mathrm{CV}R_{CO_{2}}$ mean response time when included as a covariate in our ANOVA model. Accordingly, one may speculate that $\mathrm{CV}R_{CO_{2}}$ during adolescence is influenced by the sex‐specific and divergent interaction of vasoactive gonadal hormones and extra‐cranial arterial wall structure. However, the inclusion of resting ICA_Diam_ and ICA_SR_ as covariates did not appear to influence the ANOVA model for steady‐state $\mathrm{CV}R_{CO_{2}}$ or the mean response time. Nonetheless, sex differences in $\mathrm{CV}R_{CO_{2}}$ may be due to the Douglas bag method of inducing hypercapnia, invoking sex‐specific differences in the relationship between ventilation, the $P_{\mathrm{aC}O_{2}}$–$P_{CO_{2}}$ gradient, haematological characteristics of oxygen carrying capacity and cerebrovascular haemodynamics (Fisher, 2016; Tallon et al., 2020).

Unlike gCBF, there was no influence of training status on steady‐state $\mathrm{CV}R_{CO_{2}}$ in pre‐ or post‐PHV youth. Several studies utilising TCD to measure $\mathrm{CV}R_{CO_{2}}$ have demonstrated a positive effect of endurance training on $\mathrm{CV}R_{CO_{2}}$ (Bailey et al., 2013; Barnes et al., 2013; Murrell et al., 2013). However, Barnes et al. (2013) only showed a positive relationship between cardiorespiratory fitness and $\mathrm{CV}R_{CO_{2}}$ in older adults, while Murrell et al. (2013) only observed a change in $\mathrm{CV}R_{CO_{2}}$ in young adults during submaximal exercise, but not at rest. Therefore, steady‐state $\mathrm{CV}R_{CO_{2}}$ may be unaffected by endurance training and cardiorespiratory fitness during youth and young adulthood, and instead, may only be modifiable in older adults following age‐related declines in gCBF and cerebrovascular function. That said, Dubose et al. (2022) demonstrated a quadratic relationship between steady‐state $\mathrm{CV}R_{CO_{2}}$ and cardiorespiratory fitness in older adults (DuBose et al., 2022). Therefore, the relationship between cardiorespiratory fitness and $\mathrm{CV}R_{CO_{2}}$ is complex and perhaps non‐linear across the fitness spectrum, which may have implications for its utility as a measure of cerebrovascular function in different cohorts. Conversely, the $\mathrm{CV}R_{CO_{2}}$ mean response time was faster in endurance trained pre‐PHV youth compared to untrained counterparts, suggesting endurance training in pre‐PHV youth develops $\mathrm{CV}R_{CO_{2}}$ kinetics closer to the post‐PHV phenotype. Furthermore, the significant relationship between cardiorespiratory fitness and $\mathrm{CV}R_{CO_{2}}$ mean response time in pre‐PHV youth reinforces that endurance training mediated the faster response in trained youth. Conversely, there were no training‐related differences in mean response time with endurance training in the older groups, suggesting that the adaptation of cerebrovascular function is more readily attainable during early adolescence.

### 4.3 Implications

Chronic endurance training in adults modulates brain blood flow (Ainslie et al., 2008; Alfini et al., 2019; Bailey et al., 2013; Chapman et al., 2013; Kleinloog et al., 2019; Tarumi et al., 2013; Thomas et al., 2013) and cerebrovascular reactivity to CO_2_ (Bailey et al., 2013; Barnes et al., 2013; DuBose et al., 2022; Murrell et al., 2013). Endurance training can improve cerebral perfusion, nutrient delivery and by‐product removal which, in turn, is likely to provide neuroprotective benefits across adulthood. Our findings demonstrate that exercise training‐mediated adaptations in gCBF and $\mathrm{CV}R_{CO_{2}}$ kinetics are feasible far earlier than the pathogenesis of neurocognitive disease. Future studies should attempt to identify if the exercise‐induced adaptations can be optimised with different endurance training programmes (i.e. moderate intensity aerobic work, high intensity interval training or resistance training). Moreover, using multi‐modal imaging techniques to confirm whether exercise‐mediated adaptations in gCBF and $\mathrm{CV}R_{CO_{2}}$ are linked to regional brain blood flow and neurocognitive test performance in pre‐ and post‐PHV youth may help inform the role exercise plays in optimising cerebrovascular development and long‐term health.

### 4.4 Methodological considerations

There are a few limitations that must be considered in the current study. First, we chose to implement a fixed bolus of 6% inspired CO_2_ over 4 min to manipulate $P_{\mathrm{aC}O_{2}}$, which in turn was indirectly monitored via $P_{\mathrm{ETC}O_{2}}$. There are several different approaches to invoking hypercapnia for the assessment of $\mathrm{CV}R_{CO_{2}}$, including rebreathing techniques, a fixed bolus of inspired CO_2_, computerised prospective targeting of $P_{\mathrm{ETC}O_{2}}$ and $P_{\mathrm{ET}O_{2}}$, and computerised dynamic $P_{\mathrm{ETC}O_{2}}$ and $P_{\mathrm{ET}O_{2}}$ clamping (Fierstra et al., 2013; Hoiland et al., 2019), which invoke subtle differences in the chemoreceptor, ventilatory and vasomotor stimuli (Brothers et al., 2014; Hoiland et al., 2019). We chose to implement this methodology due to its previous successful implementation in youth and its ability to distinguish child–adult differences in $\mathrm{CV}R_{CO_{2}}$ kinetics (Tallon et al., 2020). Furthermore, although this approach does not allow for precise control of $P_{\mathrm{ETC}O_{2}}$ or $P_{\mathrm{ET}O_{2}}$ or inter‐individual variability in the $P_{\mathrm{aC}O_{2}}$ to $P_{\mathrm{ETC}O_{2}}$ gradient, $P_{\mathrm{ETC}O_{2}}$ is frequently used as a surrogate for $P_{\mathrm{aC}O_{2}}$ when studying the cerebral blood flow response to hypercapnia (Al‐Khazraji et al., 2021, 2019; Coverdale et al., 2015; Ellis et al., 2017; Leung et al., 2016; Peltonen et al., 2016; Tallon et al., 2022) and has a strong relationship with $P_{\mathrm{aC}O_{2}}$ in non‐ventilated children (Berkenbosch et al., 2001; Nosovitch et al., 2002). Thus, our data still provide novel insight into the influence of maturity and training status on the cerebral blood flow response to hypercapnia. Additionally, a strength of our CO_2_ manipulation protocol was that the end‐tidal and blood pressure stimulus was similar across groups, and therefore we are confident in our steady‐state $\mathrm{CV}R_{CO_{2}}$ and mean response time results. Second, we chose not to control laboratory visits for menstrual cycle phase in female participants, as the main aim of this study was to investigate the influence of training status on gCBF and $\mathrm{CV}R_{CO_{2}}$ at different maturity stages in males and females. As such, there will be normal biological variability in circulating sex‐specific hormones between pre‐ and post‐PHV groups, as well as males and females, irrespective of cycle phase (Gurvich et al., 2018). Finally, we distinguished participants as being pre‐ or post‐PHV, which is a measurement of somatic maturation. There is evidence that the accuracy of cross‐sectional predictions of an individual's somatic maturity may be influenced by biological sex (Malina et al., 2021) and training status (Nariyama et al., 2001). Furthermore, different facets of maturation which display inter‐individual variability in their developmental trajectory (biological, sexual, social, etc.) may well impact on cerebral metabolism and cerebrovascular function. However, we chose PHV to quantify categorical stages of somatic maturity for the current study with a distinct cut‐off of ±0.5 years to classify pre‐ and post‐PHV youths, due to the non‐intrusive nature and the reliability of PHV in quantifying categorical stages of somatic maturity across a cohort (Boeyer et al., 2020; Kozieł & Malina, 2018; Mirwald et al., 2002).

### 4.5 Conclusions

Our novel findings demonstrate that cerebral blood flow is elevated in endurance trained youth, and particularly post‐PHV males. However, the kinetic response to hypercapnia is faster in pre‐, but not post‐PHV trained youth when compared to their untrained counterparts. Furthermore, cardiorespiratory fitness was significantly associated with cerebral blood flow in post‐ but not pre‐PHV youth, while there was a significant relationship between cardiorespiratory fitness and cerebrovascular reactivity to CO_2_ in pre‐ but not post‐PHV youth. Therefore, higher cardiorespiratory fitness can elevate cerebral blood flow in adolescent youth, while endurance exercise training is associated with faster cerebrovascular reactivity to CO_2_ during childhood. Accordingly, our findings highlight the positive role of endurance training on cerebrovascular function across youth.

## AUTHOR CONTRIBUTIONS

Jack S. Talbot, Jon L. Oliver, Rhodri S. Lloyd, Philip N. Ainslie, Ali M. McManus and Mike Stembridge contributed to the conception and design of the study. All authors were involved in the acquisition, analysis or interpretation of data for the work. All authors were involved in drafting the work or revising it critically for important intellectual content. Additionally, all authors approved the final version of the manuscript and agree to be accountable for all aspects of the work in ensuring that questions related to the accuracy or integrity of any part of the work are appropriately investigated and resolved. All persons designated as authors qualify for authorship, and all those who qualify for authorship are listed.

## CONFLICT OF INTEREST

The authors have no competing interests to declare.

## Data Availability

The data that support the findings of this study are available from the corresponding author upon reasonable request.
